# Which environments matter in studies of early life developmental plasticity?

**DOI:** 10.1093/emph/eox024

**Published:** 2018-02-05

**Authors:** Christopher W Kuzawa

**Affiliations:** Department of Anthropology, Northwestern University, Evanston, IL 60201, USA

In 1989, the British epidemiologist David Barker and his colleagues [1] reported intriguing findings from the UK showing that risk for heart disease was highest among people who were born small and thin. This ran counter to conventional wisdom regarding the role of nutrition and weight in cardiovascular diseases, which were classically viewed as ‘diseases of excess’. Research on animal models soon confirmed that restricting nutrient delivery to the fetus induces a similar constellation of adult disease outcomes. This work left little doubt that gestational nutrition and other prenatal factors influence one’s risk for developing many chronic degenerative diseases, which forced a rethink of their fundamental causes.

To researchers in evolutionarily focused fields, the finding that early life undernutrition heightens one’s risk for diseases related to adult overweight looked like developmental plasticity gone awry [2]: the findings suggested that nutrient set points might be calibrated to maternal nutrient delivery *in utero* and that this could then backfire if future environments changed abruptly, as is occurring with the rapid lifestyle and dietary changes that are sweeping much of the globe. Bateson [3] described such effects as akin to the mother sending a ‘weather forecast’, a concept later rebranded in the more general concept of a ‘predictive adaptive response’ [4].

This idea has intuitive appeal. Similar examples of anticipatory plasticity have been noted in other species. For instance, the tadpoles of spadefoot toads will speed through morphogenesis in response to a drying pond, and rodents that live in the arctic can use maternal melatonin to sense the season in which they will be born and adjust growth rate and the timing of reproduction accordingly. Although such species provide clear evidence for predictive developmental adaptation, there is a problem with extrapolating to humans from such examples: these are short-lived species for whom early ecological cues carry high-fidelity information about the environment an adult will encounter. What happens when lifespans are long-spanning not one or two seasons, but 70 or 80 years? Is it realistic that a long-lived species like humans could find an adaptive advantage in predicting adult environments from gestational conditions [5]?

In their insightful review appearing in *EMPH*, Lea and colleagues make a compelling case for the need to combine the conceptual and methodological tools of evolutionary biology and health-oriented research to help address these questions. They rightly note that attempts to test this idea in humans have generally yielded negative findings and that there is little evidence for long-term developmental prediction in species with life history characteristics more akin to humans. In the authors’ own work among the baboons of Amboseli, who have been followed longitudinally for decades, animals born in difficult times always fair more poorly as adults, even when adult conditions are poor. This runs counter to the hypothesis of predictive adaptation, which leads to the expectation that experiencing a nutritionally restrictive early environment will better equip an individual to handle future nutrient restriction. The few explicit tests of this model in humans have similarly failed to provide evidence for long-term developmental adaptation. I appreciate the balance of this review, and agree with the authors’ assessment of the state of this hypothesis and its applicability to humans.

Lea and colleagues’ discussion of genetic and molecular approaches is also a useful addition to the field. Given the many biological systems involved with developmental plasticity, the contributing pathways are likely to be especially complex and hard to anticipate. This points to the need for, among other things, discovery-based approaches that are hypothesis-free and avail of genome-wide genetic and epigenetic data, and other bioinformatics-driven approaches, to uncover underlying mechanisms.

Such approaches focus on uncovering how genes interact with early environments to shape outcomes (what the authors call ‘G x early E interactions’). One complicating dimension of this problem lies in defining what constitutes an early environment. This is often underappreciated, and indeed is not discussed at length by Lea and colleagues. In mammals, the age of greatest plasticity is typically during gestation, when plasticity impacts numerous traits like metabolism, growth, stress physiology and immunity. The fetus does not respond to the environment itself during gestation but rather to nutrients, hormones and other signals received from the mother’s body. This gestational milieu is maintained in a relatively stable state by the mother’s homeostatic biology, as illustrated by the example of nutrient delivery. When a mother stops eating, under most conditions her body maintains constant availability of circulating glucose, which directly benefits the fetus. This is achieved by mobilizing glycogen stores, stored fats and amino acids. Her muscle also stops responding fully to insulin, shunting glucose to the fetus. As a result of these homeostatic processes, any nutritional stress that a mother experiences may be invisible to her fetus, and this buffering capacity appears to be particularly effective in larger and longer-lived species like humans [6]. In human populations, maternal buffering helps explain why nutritional stress often has minimal impacts on birth weight, and similarly why pregnancy dietary supplements typically have negligible positive effects on birth outcomes [7].

Given this, we need to understand the extent to which the homeostatic set points that maintain stability in the gestational environment are altered in response to maternal experience—and if so, to what factors and on what time scales. Although offspring birth weight does not respond acutely to most changes in a mother’s diet during pregnancy, birth weight *is* predicted by the mother’s weight and body composition *prior* to pregnancy [8]. In other words, offspring birth weight is predicted by a mother’s long-term energy balance—a clear reflection of ecological quality—in the years preceding pregnancy. Studies also point to a mother’s own early developmental nutrition as an important predictor of offspring birth weight [9, 10]. In light of these findings, it seems likely that the human fetus is not tracking what its mother eats but rather what she *ate* – over a longer timeframe. This suggests that the mother’s body conveys integrated information about nutritional experiences across her lifecycle, information that could provide a reliable cue of long-term local conditions for a long-lived species [5]. Although this hypothesis remains to be tested, studies that simply assume that organisms will adapt to short-term external environmental conditions at the time of birth or during pregnancy may be missing the mark. Historical conditions that may affect the gestational environment should be considered in studies examining predictive adaptation in long-lived species like humans or baboons.

Lea and colleagues have made an elegant call for the need to think in a more sophisticated way about the biological complexities of developmental plasticity, including application of current molecular and genomic methods. I would only add that we should take a similarly sophisticated approach to measuring and modeling the environment— another dimension of great complexity. Research over several decades has made it clear that the periods of greatest plasticity in mammals occur at an age when resources are derived from maternal physiology and metabolism, not directly from the external environment. The questions that we ask about plasticity should reflect that reality. To understand the genetic and developmental pathways that link fetal experiences with later biology and health, we need to understand the historical and ancestral inputs that influence gestational biology, and by extension, the developmental trajectory of the next generation.

**Conflict of interest:** None declared.
